# Artificial Intelligence–Based Framework for Analyzing Health Care Staff Security Practice: Mapping Review and Simulation Study

**DOI:** 10.2196/19250

**Published:** 2021-12-22

**Authors:** Prosper Kandabongee Yeng, Livinus Obiora Nweke, Bian Yang, Muhammad Ali Fauzi, Einar Arthur Snekkenes

**Affiliations:** 1 Department of Information Security and Communication Technology Norwegian University of Science and Technology Gjovik Norway

**Keywords:** artificial intelligence, machine learning, health care, security practice, framework, security, modeling, analysis

## Abstract

**Background:**

Blocklisting malicious activities in health care is challenging in relation to access control in health care security practices due to the fear of preventing legitimate access for therapeutic reasons. Inadvertent prevention of legitimate access can contravene the availability trait of the confidentiality, integrity, and availability triad, and may result in worsening health conditions, leading to serious consequences, including deaths. Therefore, health care staff are often provided with a wide range of access such as a “breaking-the-glass” or “self-authorization” mechanism for emergency access. However, this broad access can undermine the confidentiality and integrity of sensitive health care data because breaking-the-glass can lead to vast unauthorized access, which could be problematic when determining illegitimate access in security practices.

**Objective:**

A review was performed to pinpoint appropriate artificial intelligence (AI) methods and data sources that can be used for effective modeling and analysis of health care staff security practices. Based on knowledge obtained from the review, a framework was developed and implemented with simulated data to provide a comprehensive approach toward effective modeling and analyzing security practices of health care staff in real access logs.

**Methods:**

The flow of our approach was a mapping review to provide AI methods, data sources and their attributes, along with other categories as input for framework development. To assess implementation of the framework, electronic health record (EHR) log data were simulated and analyzed, and the performance of various approaches in the framework was compared.

**Results:**

Among the total 130 articles initially identified, 18 met the inclusion and exclusion criteria. A thorough assessment and analysis of the included articles revealed that K-nearest neighbor, Bayesian network, and decision tree (C4.5) algorithms were predominantly applied to EHR and network logs with varying input features of health care staff security practices. Based on the review results, a framework was developed and implemented with simulated logs. The decision tree obtained the best precision of 0.655, whereas the best recall was achieved by the support vector machine (SVM) algorithm at 0.977. However, the best F1-score was obtained by random forest at 0.775. In brief, three classifiers (random forest, decision tree, and SVM) in the two-class approach achieved the best precision of 0.998.

**Conclusions:**

The security practices of health care staff can be effectively analyzed using a two-class approach to detect malicious and nonmalicious security practices. Based on our comparative study, the algorithms that can effectively be used in related studies include random forest, decision tree, and SVM. Deviations of security practices from required health care staff’s security behavior in the big data context can be analyzed with real access logs to define appropriate incentives for improving conscious care security practice.

## Introduction

### Background

Unlike other sectors, the health care sector cannot afford to implement stricter control for accessing sensitive health care information for therapeutic purposes. Despite the recognized need to provide tighter security measures in controlling access, there is also the need to strike a balance for allowing legitimate access to health care data for therapeutic reasons [1,2]. In access control management in health care, access to personal health data and personal data filing systems for therapeutic purposes must be granted following a specific decision based on “the completed or planned implementation of measures for the medical treatment of the patient” [3]. Therefore, access must only be granted to those with official needs [3,4]. While providing restrictions against unauthorized access, there are some provisions for following the availability trait of the confidentiality, integrity, and availability (CIA) triad during emergency situations. These include the provision for self-authorization. Self-authorization, or “break-the-glass,” is a “technical measure which has been established for health personnel to be able to gain access to personal health data and personal data as and when necessary” [1]. However, access through self-authorization must be verified for abuse, and clear misuse must be followed up as a data breach [3,5].

The challenge remains in detecting misuse over a broad range of access [1,2]. A broad range of access via self-authorization results in tones of variant data known as “big data” [6], making it complex to manually determine legitimate access. However, in light of the recent increase in data breaches within health care, it has become necessary to adopt state-of-the-art methods to determine anomalous access. In the Healthcare Security Practice Analysis, Modeling, and Incentivization (HSPAMI) project [7], data-driven and artificial intelligence (AI) approaches were identified and adopted to aid in modeling and analyzing health care staff’s security practices in their access control logs [7]. AI is based on algorithms in computer science that can be used for analyzing complex data to draw meaningful patterns and relationships toward decision making [8]. The aim of this study was to understand anomaly practices in health care in the context of big data and AI, and to determine the security practice challenges often faced by health care workers while performing their duties. The results will provide knowledge to serve as a guide for finding better approaches to security practice in health care. However, there are different types of data sources and AI methods that can be used in this approach [7]. We therefore adopted a review methodology to first detail various types of dimensions, including the data sources and AI methods, which can be adopted in related studies.

According to Verizon, the health care sector globally experienced approximately 503 data breaches in 2018, which resulted in the compromise of up to 15 million records [4,9]. This figure was triple the number of data breaches recorded in 2017. In addition, the number of records compromised within the health care sector in 2019 far exceeded that recorded in 2018 [9]. Unfortunately, more than half of these data breaches were perpetuated by insiders [9]. The report opined that approximately 83% of the adversaries were motivated by financial gains, 3% were due to convenience, 3% were due to grudges, and 2% were a result of industrial espionage. The current situation implies that the number of data breaches within the health care sector has surpassed that of the financial sector and almost equals those of other public sectors.

This situation has raised concerns among relevant stakeholders, and many are wondering the reasons behind the spike in the number of data breaches within the health care sector. Some of these reasons can be easily deduced because health care data have economic value and as such represent a possible target for malicious actors [10,11]. Moreover, health care data have scientific and societal value that makes them very attractive for cyber criminals. In fact, Garrity et al [12] indicated that patient medical records are sold for approximately US $1000 on the dark web. Another reason for data breaches within health care is the lack of health care personnel. The few health care personnel are more interested in their core health care duties and have little time to handle health care information security issues. This situation provides cyber criminals with the opportunity to exploit health care systems.

Although there have been improvements in technical measures, such as firewalls, intrusion detection and prevention systems, antivirus software, and security governance configurations, the development of a “human firewall” has not been considered [13,14]. The “human firewall” refers to the information security conscious care behavior of insiders [15]. However, this concept has not received equal attention as devoted to technical measures, and thus cyber criminals seek to exploit it for easy access [16]. Health care insiders have access privileges that enable them to provide therapeutic care to patients; however, through errors or deliberate actions, they can compromise the CIA of health care data. It is also possible for an attacker to masquerade as an insider to compromise health care data through social engineering and other methods [17,18].

Access control mechanisms within the health care sector are usually designed with a degree of flexibility to facilitate efficient patient management [19]. Even though such design considerations are vital and can meet the availability attribute of the CIA, they make health care systems vulnerable. This is because flexibility can be abused by insiders [20]. In addition, an attacker who could obtain an insider’s access privilege can exploit this flexibility to have broader access. A successful data breach could have many consequences such as denial of timely medical services, corrosion of trust between the patient and health care providers, breaches to an individual’s privacy [21], and huge fines to health care providers by national and international regulatory bodies. The general objective of this study was to determine an effective way of modeling and analyzing health care logs. A review was first performed to retrieve appropriate data sources and their features in addition to identifying the AI methods that can best be used to determine irregularities in security practices among health care workers.

### Prior Studies

The security practices of health care staff include how health care professionals respond to security controls and measures for achieving the CIA goals of health care organizations [2,4,5]. Health care professionals are required to conduct their work activities in a security-conscious manner to maintain the CIA of the health care environment [3]. For instance, borrowing access credentials could jeopardize the purpose of access control for authorized users and legitimate access. Additionally, the inability to understand social engineering scammers’ behavior can lead to health care data breaches [7].

Various approaches can be adopted to observe, model, and analyze health care professionals’ security practices. A perception and sociocultural context can be adopted by analyzing the security perception, and social, cultural, and sociodemographic characteristics of health care staff in the context of their required security practices [7,22]. In addition, an attack-defense simulation can be used to measure how health care staff understand social engineering–related tricks. Furthermore, a data-driven approach with AI methods could be adopted to understand the security behavior of each health care professional in the context of big data, since AI is most appropriate for analyzing complex data sets with high volume, variety, velocity, and veracity [8]. The findings can then help decision makers to introduce appropriate incentive methods and solve issues that hinder sound information security practice toward enhancing conscious care behavior.

Advances in computational and data science, along with engineering innovations in medical devices, have prompted the need for the application of AI in the health care sector [23-25]. This has the potential to improve health care delivery and revolutionize the health care industry. AI can be referred to as the use of complex algorithms and software to imitate human cognitive functions [24-26]. AI involves the application of computer algorithms in the process of extracting meaning from complicated data and making intelligent decisions without direct human input [24,25]. AI is increasingly impacting every aspect of our lives, and the health care sector is no exception. In recent years, the health care sector experienced massive AI deployments in the bid to improve overall health care delivery. We here rely on the classification of the application of AI in health care described by Wahl et al [27] to briefly discuss the deployment of AI in health care.

According to Wahl et al [27], the deployment of AI in the health care sector has been classified to include expert systems, machine learning, natural language processing, automated planning and scheduling, and image and signal processing [27]. Expert systems are AI programs that have been trained with real cases to execute complicated tasks [28]. Machine learning employs algorithms to identify patterns in data and learn from them, and its applications can be grouped into three categories: supervised learning, unsupervised learning, and reinforcement learning [25,27]. Natural language processing facilitates the use of AI to determine the meaning of a text by using algorithms to identify keywords and phrases in natural language. Automated planning and scheduling is an emerging field in the use of AI in health care that is concerned with the organization and prioritization of the necessary activities to obtain the desired aim [27]. Image and signal processing involves the use of AI to train information extracted from a physical occurrence (images and signals) [27].

The common characteristic of all these applications is the utilization of massive data that are being generated in the health care sector to make better informed decisions. For instance, the collection of data generated by health care staff has been used for disease surveillance, decision support systems, detecting fraud, and enhancing privacy and security [29]. In fact, the code of conduct for the Norwegian health care sector requires the appropriate storage and protection of access logs of health care information systems for security reasons [3]. Health care staff’s access to the network or electronic health records (EHR) leaves traces of their activities, which can be logged and reconstructed to form their unique profiles [3,4]. Therefore, appropriate AI methods can be used to mine such logs to determine the unique security practices of health care staff. Such findings can support management in adapting suitable incentivization methods toward improving security-conscious care behavior in health care. Therefore, the aim of this study was to explore the appropriate AI methods and data sources that can be used to observe, model, and analyze the security practices of health care staff.

HSPAMI is an ongoing research project with one aspect involving the modeling and analysis of data with AI methods to determine the security practices of health care staff toward improving their security-conscious care behavior. In analyzing health care–related data, there is a need to consider details of the methods and data sources in view of the unique and critical nature of the sector. In a related study, Walker-Roberts et al [30] performed a systematic review of “the availability and efficacy of countermeasures to internal threats in health care critical infrastructure.” Among various teams, few machine learning methods were identified to be used for intrusion detection and prevention. The methods that were identified are Petri net, fuzzy logic, k-nearest neighbor (KNN), decision tree (RADISH system) [30-32], and inductive machine learning methods [30,31,33]. In a similar way, Islam et al [34] performed a systematic review on data mining for health care analytics. Categories such as health care subareas; data mining techniques; and the types of analytics, data, and data sources were considered in the study. Most of the data analysis was focused on clinical and administrative decision-making. The data sources were mostly human-generated from EHRs. Gheyas et al [35] also explored related methods in their systematic review and meta-analysis [35].

Even though the studies of Walker-Roberts et al [30] and Islam et al [34] were in the health care context, details of the algorithms and data sources were not considered. For instance, the features of the data sources and algorithm performance methods were not deeply assessed in their studies. Additionally, these studies were general and not specific to health care [35,36], and therefore the unique challenges within the health care environment were not considered. To this end, this study explored AI methods and data sources in health care that can be efficiently used for modeling and analyzing health care professionals’ behavior. The terms “health care professionals” and “health care staff” are used interchangeably in this paper, which include, but are not limited to, nurses, physicians, laboratory staff, and pharmacies who access patient records for therapeutic reasons.

### Scope, Problem Specification, and Contribution

Following the recent increase in data breaches in health care, our research group is working on the HSPAMI project, which was initiated to measure the information security practice level of health care staff [7,22]. The results will help provide better approaches for incorporating conscious care behavior among health care staff. The HSPAMI project has already identified various approaches to include psychosociocultural context attack and defense simulations in a social engineering context along with data-driven AI approaches [7].

The main goal is to demonstrate how health care security practices can be analyzed to determine anomalous and malicious activities in the context of data-driven and AI approaches. Therefore, the specific objectives of this study were to identify, assess, and analyze the state-of-the-art data-driven attributes and AI methods along with their design strategies and challenges. A framework for analyzing health care security practice in the context of data-driven and AI methods was also developed and evaluated. The broad goal was to enable analysis of real logs of health care professionals’ security practices in the context of big data and human-generated data logs. Therefore, the psychosociocultural context and attack-defense simulations are beyond the scope of this paper.

Some details of data sources and AI methods that can be used in this study were not provided in previous related work [30-34], which raised several questions for our research: Among the various data sources that are generated by health care staff, which is the most appropriate to be used in analyzing the security practice? Which AI methods have been pinpointed to be suitable for use in modeling and analyzing health care security practice? What evaluation techniques are most appropriate in this context, and how were these methods adjusted to curtail biases amid various access points, such as self-authorization during emergency care scenarios and the busy schedules of health care staff? To answer these questions, we first performed a mapping review [37] toward identifying, modeling, and analyzing health care staff–generated access logs and AI methods to enhance security practice. This work represents an extended version of our previous work, with the additions being a design and framework evaluation.

## Methods

### Literature Review

Various types of systematic studies exist [38-41], including a systematic mapping study, scoping review, and systematic literature review. Systematic mapping studies review topics with a broader scope by categorizing the identified research articles into specific areas of interest. Systematic mapping studies have general research questions with the objective to determine research trends or the state-of-the-art studies. By contrast, the objective of a systematic literature review is to accumulate data and therefore has a more specific research focus. To this end, a systematic mapping study was adopted in this work [38,39]. Based on the results, we developed a framework that was evaluated with simulated log data.

Although we did not restrict the article search to a specific time frame, we performed the literature search between June 2019 and December 2019 with the Google Scholar, Science Direct, Elsevier, IEEE Explore, ACM Digital, Scopus, Web of Science, and PubMed databases. Different keywords were used, including “healthcare,” “staff,” “employee,” “information security,” “behavior,” “practice,” “threat,” “anomaly detection,” “intrusion detection,” “artificial intelligence,” and “machine learning.” To ensure a high-quality searching approach, the keywords were combined using the Boolean functions “AND,” “OR,” and “NOT.” For instance, the following search string was generated in PubMed:

((Intrusion[All Fields] AND Detection[All Fields]) OR (Anomaly[All Fields] AND Detection[All Fields])) AND (“health”[MeSH Terms] OR “health”[All Fields]) AND ((“artificial intelligence”[MeSH Terms] OR (“artificial”[All Fields] AND “intelligence”[All Fields]) OR “artificial intelligence”[All Fields]) OR (“machine learning“[MeSH Terms] OR (“machine”[All Fields] AND “learning”[All Fields]) OR “machine learning”[All Fields])) AND (“information”[All Fields] AND Security[All Fields]) AND ((“behavior”[All Fields] OR “behavior”[MeSH Terms] OR “behavior”[All Fields]) OR “practice”[All Fields]).

Peer-reviewed articles were considered. The inclusion and exclusion criteria were developed based on the objective of the study and through rigorous discussions among the authors.

**Figure 1:**
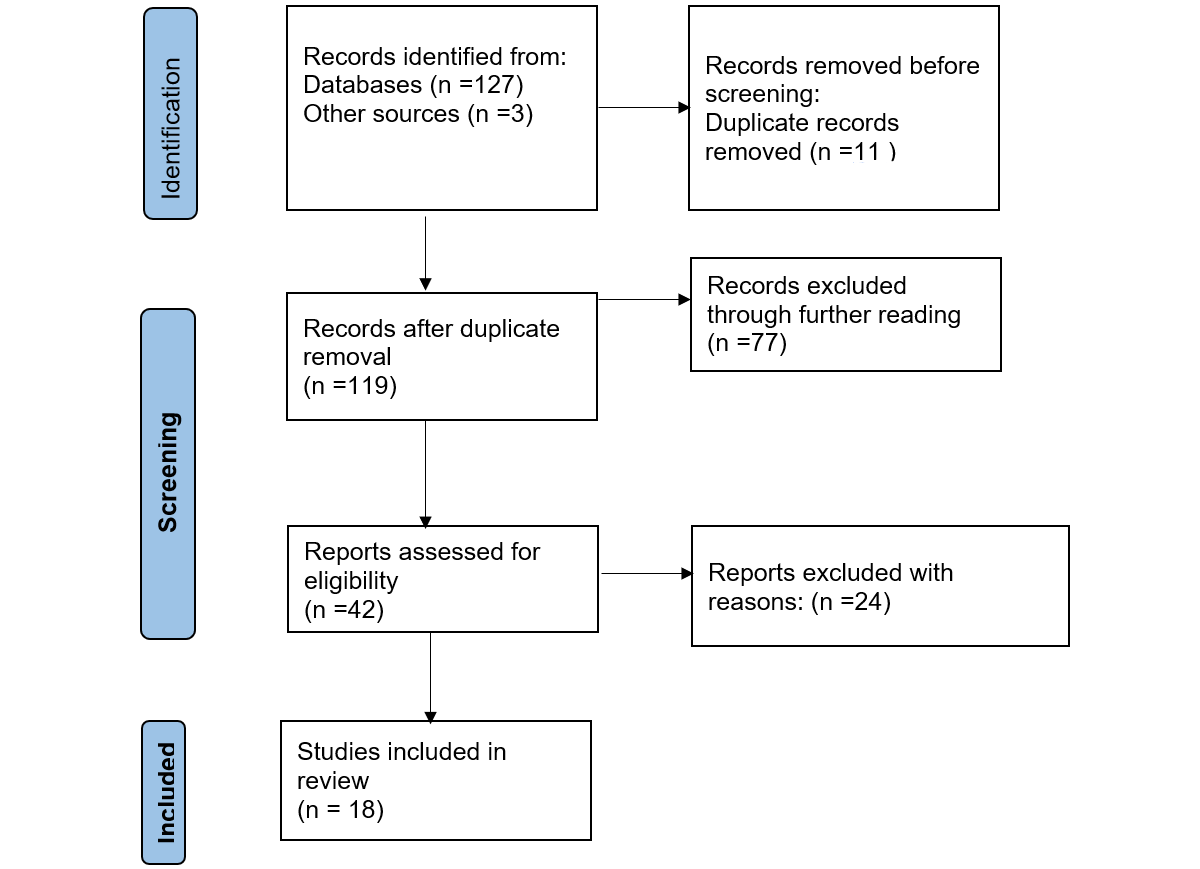
Flowchart of the systematic review process.

Basic selection was performed by initially skimming through the titles, abstracts, and keywords to retrieve records that were in line with the inclusion and exclusion criteria. Duplicates were filtered out, and articles that seemed relevant, based on the inclusion and exclusion criteria, were fully read and evaluated. Each of the authors independently read and assessed all of the selected articles and judged either to be included or excluded. Using the inclusion and exclusion criteria as a guideline, discrepancies were discussed and resolved among the authors. Other appropriate articles were also retrieved using the reference list of accepted literature. Figure 1 shows the PRISMA (Preferred Reporting Items for Systematic Reviews and Meta-Analysis) [42] flowchart of article screening and selection.

### Inclusion and Exclusion Criteria

For an article to be included in the review, it had to be related to anomaly detection or intrusion detection in health care using AI methods with health care professional–generated access log data or patterns. Any other article outside the above scope (such as articles related to medical cyber-physical devices, body area networks, and similar), along with articles published in languages other than English, were excluded.

### Data Collection and Categorization

The data collection and categorization methods were developed based on the study objective, and thorough literature reviews and discussions among the authors. The categories were defined exclusively to assess, analyze, and evaluate the study objectives, which are summarized in Table 1.

**Table 1:** Data categories and their exclusive definitions.

Category	Definition	Examples
Type of AI^a^ method	Explicit machine learning methods	Support vector machine, Bayesian network
Type of input	Features used by the algorithm	Access location, time, failed login attempts
Input sources	Type of access log data used in the study	Browser history, network logs, host-based activity logs, EHR^b^ logs
Data format, type, size, and data source	File formats	XML, comma separated value (CSV)
Input preprocessing	Defines how the data were preprocessed and how missing and corrupted input data were handled	Structured vs unstructured
Security failures	Context in which the algorithm was implemented	Intrusion or anomaly detection
Ground truth	Type of training set used in training the model	Login and logout time, average number of patient records accessed
Privacy approach	Defines the privacy method used to safeguard the privacy rights of individuals who contributed to the data source	Message Digest 5 (MD5), Secure Hash Algorithm (SHA)-3
Performance metrics or evaluation criteria	Measures used to assess the accuracy of the study	Specificity, sensitivity, receiver operating characteristic curve
Nature of data sources	Specifies whether the data used were synthetic or real data	Real data, simulated data

^a^AI: artificial intelligence.

^b^EHR: electronic health record.

### Literature Evaluation and Analysis

The selected articles were assessed, analyzed, and evaluated based on the categories defined in Table 1. The analysis was performed on each of the categories (eg, type of AI method, type of input, input source, preprocessing, learning techniques, performance methods) to evaluate the state-of-the-art approaches. Percentages of the attributes of the categories were calculated based on the total number of counts (n) of each type of attribute. Some studies used multiple categories; therefore, the number of counts of these categories exceeded the total number of articles of these systems presented in the study.

## Results

### Review Findings

#### Articles Retrieved

After searching the various online databases, a total of 130 records were initially identified following the guidelines of the inclusion and exclusion criteria in the reading of titles, abstracts, and keywords. A further assessment of these articles through skimming of the objective, method, and conclusion sections led to an exclusion of 77 articles that did not meet the defined inclusion criteria. After removing duplicates, 42 articles were fully read and judged. After full-text reading, a total of 18 articles were included in the study and analysis (Figure 1).

#### Algorithms

**Figure 2:**
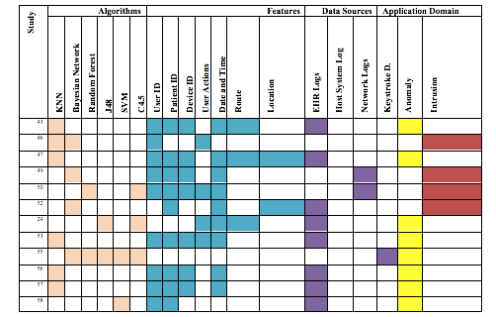
Algorithms, features, related data sources, and application domain. KNN: k-nearest neighbor; SVM: support vector machine; EHR: electronic health record.

The main findings of the reviewed articles and their related categorizations such as algorithms, features, and data sources are shown in Figure 2. The algorithms, features, data sources, and application domains were the most frequent categorizations in the review; the study column presents the sources of each of these categories.

The algorithms that were most commonly used for analyzing security practice in the review are shown in Table 2. The KNN method was the most frequently used, followed by the Bayesian network and C4.5 decision tree.

**Table 2:** Algorithms and their respective proportions among the articles included in the review (N=30).

Algorithm	Studies, n (%)	References
K-nearest neighbor	5 (17)	[43-47]
Bayesian network	4 (13)	[43,44,48,49]
Decision tree (C4.5)	3 (10)	[24,49,50]
Random forest	2 (7)	[49,50]
J48	2 (7)	[24,49]
Support vector machine	1 (3)	[49,51]
Spectral projection model	1 (3)	[47]
Principal component analysis	1 (3)	[47]
K-means	1 (3)	[52]
Ensemble averaging and a human-in-the-loop model	1 (3)	[53]
Partitioning around Medoids with k estimation (PAMK)	1 (3)	[50]
Distance-based model	1 (3)	[54]
White-box anomaly detection system	1 (3)	[55]
C5.0	1 (3)	[50]
Hidden Markov model	1 (3)	[54]
Graph-based	1 (3)	[56]
Logistic regression	1 (3)	[51]
Linear regression	1 (3)	[51]
Fuzzy cognitive maps	1 (3)	[57]

#### Features

Table 3 shows the unique features identified in the review and their respective counts and proportions. The features that were the most frequently used included user ID, date and time attribute, patient ID, and device identification.

**Table 3:** Features used in the reviewed articles (N=65).

Feature	Count, n (%)
User identification	13 (20.0)
Patient identification	11 (16.9)
Device identification	9 (13.8)
Access control	5 (7.7)
Date and time	11 (16.69)
Location	4 (6.2)
Service/route	5 (7.7)
Actions (delete, update, insert, copy, view)	3 (4.6)
Roles	3 (4.6)
Reasons	1 (1.5)

#### Data Sources

The majority of the data sources were EHR logs (11/18, 61%), followed by host-based logs (2/18, 11%), network logs (4/18, 22%), and keystroke activities (1/18, 5%).

#### Performance Methods

Table 4 shows the various types of performance methods that were identified with their respective counts and proportions; recall and receiver operating characteristic curve were the most common metrics applied, whereas F-score and root mean square error were the least commonly applied.

**Table 4:** Performance methods used in the reviewed studies (N=25).

Performance methods	Studies, n (%)
Receiver operating characteristic (ROC) curve	5 (20)
Area under ROC curve	3 (12)
Recall (sensitivity)	5 (20)
Precision	4 (16)
Accuracy	2 (8)
True negative rate (specificity)	3 (12)
F-score	2 (8)
Root mean square error	1 (4)

#### Security Failures

The studies in the review were mostly applied for anomaly detection (12/18, 67%) and malicious intrusion detection (6/18, 33%).

#### File Format

Among the 4 articles that reported the file format, 2 (50%) used comma separated values [43,52] and the other 2 (50%) used the SQL file format [55,58].

#### Ground Truth

Eight of the 18 articles included in the review reported the ground truth, which was established with similarity measures (3/8, 38%), observed practices (3/8, 38%), and historical data of staff practices (2/8, 25%).

#### Privacy-Preserving Data Mining Approach

Privacy-preserving methods adopted in the included studies were tokenization [43], deidentification [45], and removal of medical information [24].

#### Nature of Data Source

The majority of studies (15/18, 83%) used real data for analysis, with the remaining (3/18, 17%) using synthetic data.

### Framework for Analyzing Health Care Staff Security Practices

**Figure 3:**
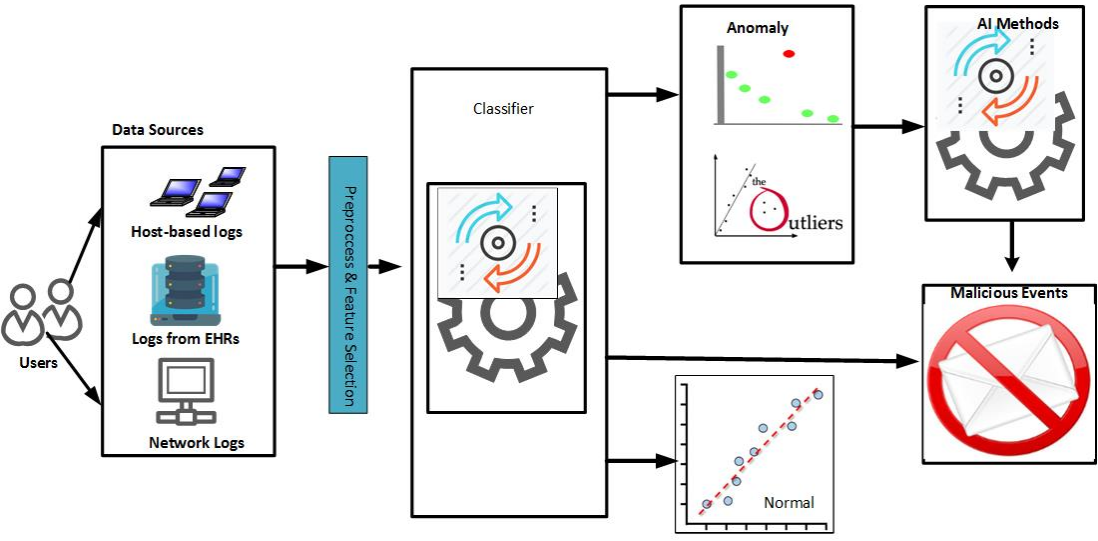
Conceptual framework for analyzing the security practices of health care staff. AI: artificial intelligence; EHR: electronic health record.

Based on the review, a conceptual framework was depicted on how data-driven and AI methods should be adopted to analyze logs of EHRs in security practice (see Figure 3). Our review indicated that a security practice analysis typically reveals the anomaly or malicious intrusion pattern of health care staff. Our model therefore has various dimensions such as data sources, preprocessing, feature extraction, the application of AI methods, and possible classes, as shown in Figure 3.

The data sources include the network, EHR, or workstation logs. These logs are generated based on health care staff activities in accessing resources such as patients, printers, medical devices, and physical security systems. The logs go through the preprocessing phase [25], such as cleaning and feature selection. The essential features are then selected with appropriate methods, including filter methods, wrapper methods, or the combined filter and wrapper approach. Having obtained the appropriate features, a machine learning method can then be created, trained, and used to detect patterns of unusual security practices. The various classes that can be deduced in this framework include normal, abnormal, significantly nonmalicious anomaly, and malicious classes. The normal class includes features that follow the flow of each established access process without access aberration. The malicious class consists of features that violate established access flow and may also include excess access, which exceeds the usual trend of users. An example includes a doctor who accesses patient records more than the average daily access, and when the access was not for therapeutic measures. The anomaly nonmalicious class includes accesses that violate the established access flow or that exceed the average daily access of the health care staff; however, in this case, the accesses were for therapeutic purposes. From the framework, three access detection methods were identified for comparison.

### Comparative Analysis of the Framework

**Figure 4:**
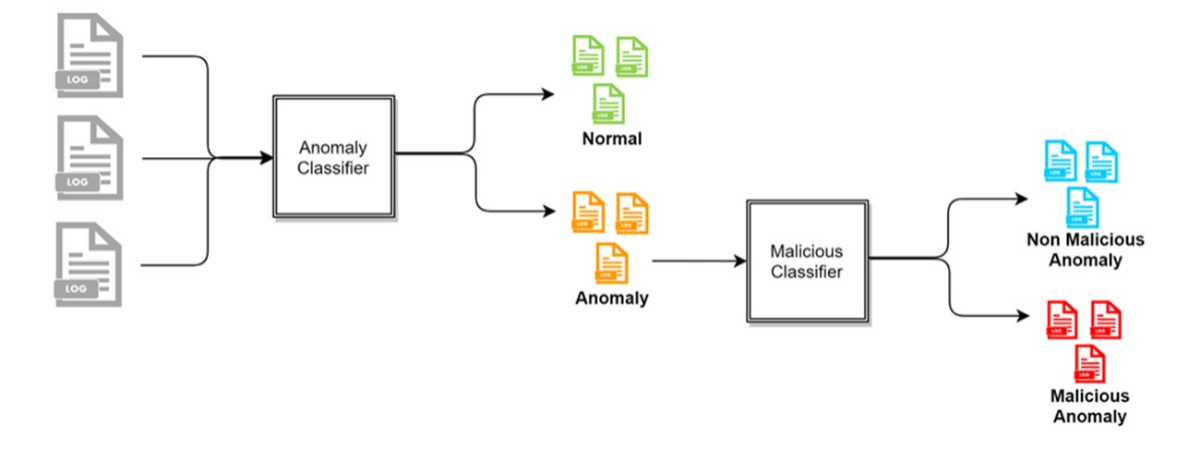
Flowchart of two-stage detection.

**Figure 5:**
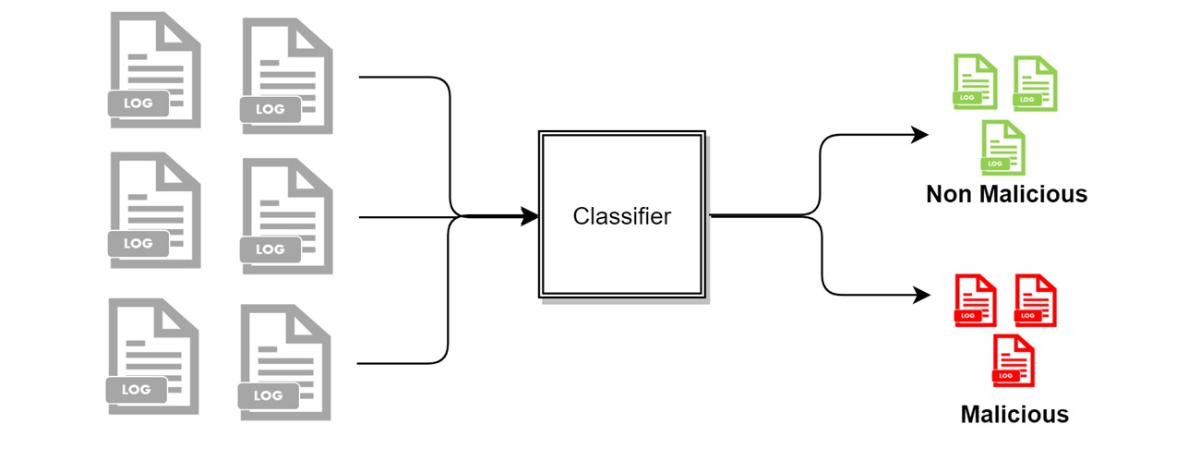
Two-class classification.

**Figure 6:**
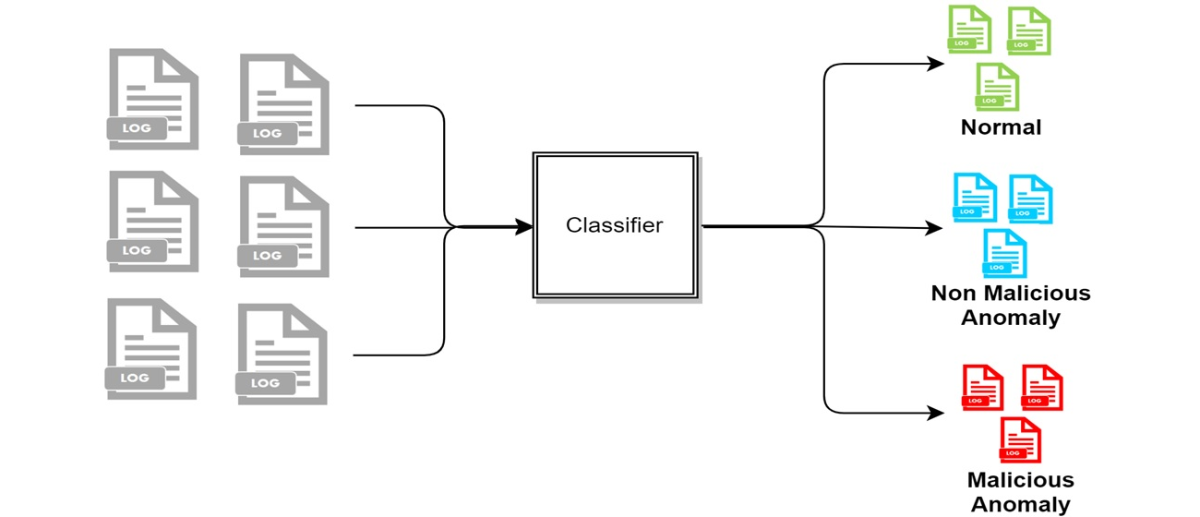
Three-class classification.

The following three access detection methods were compared: (1) two-stage classification, (2) three-class classification, and (3) two-class classification. In the two-stage classification approach, the log data are classified as normal and anomaly. The data determined in the anomaly class from the first stage are further classified into two classes: malicious and nonmalicious (Figure 4). In the three-class approach, the log data are classified into normal, nonmalicious anomaly, and malicious, as shown in Figure 5. In the two-class approach, the normal and nonmalicious anomaly data are considered as a single “nonmalicious” category. The log data are then classified into nonmalicious and malicious classes, as shown in Figure 6.

These three approaches were then compared with nine machine learning methods: multinomial naive Bayes (NB), Bernoulli NB, Gaussian NB, KNN, neural network (NN), logistic regression (LR), random forest (RF), decision tree (DT), and support vector machine (SVM).

### Simulation of EHR Logs of Health Care Staff Security Practice

**Figure 7:**
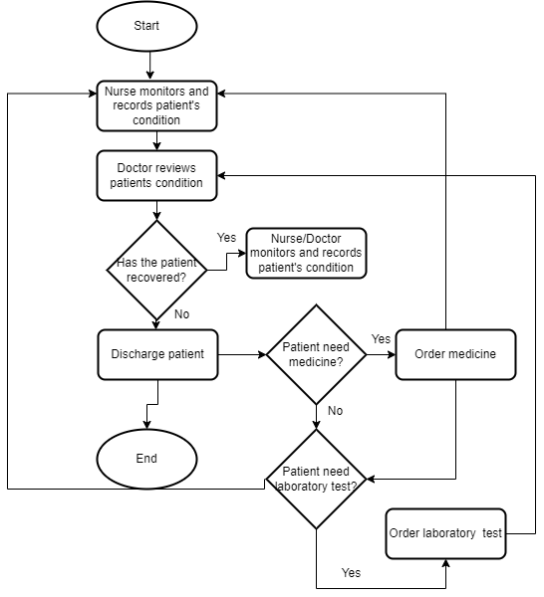
Inpatient workflow.

**Figure 8:**
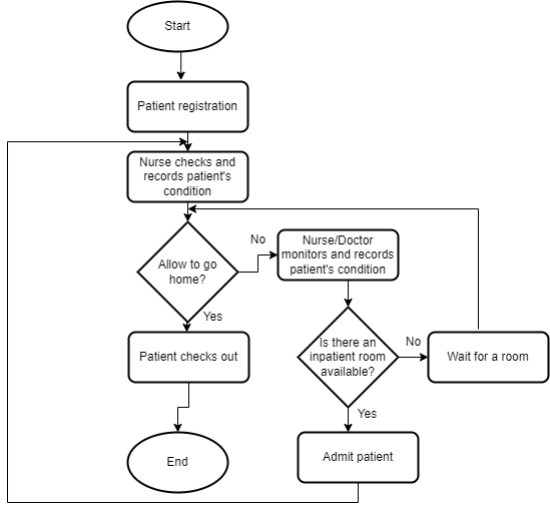
Emergency workflow.

**Figure 9:**
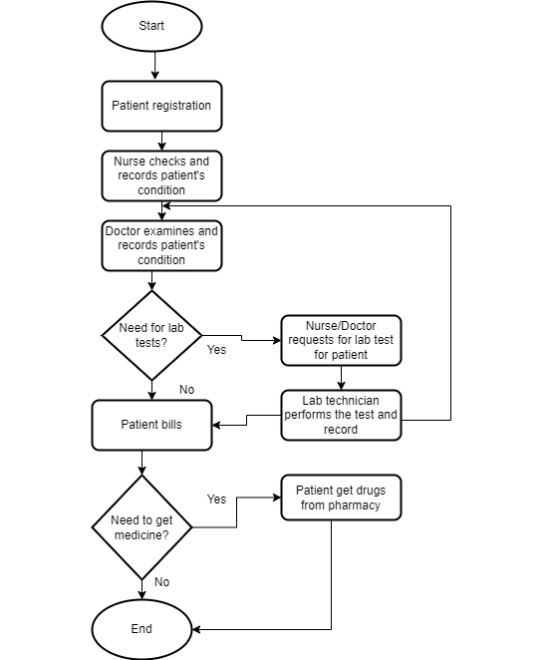
Outpatient care workflow.

The conceptual framework (Figure 3) provided direction and guidelines for effective modeling and analysis of health care staff security practices. We hence simulated 1-year access log data of a typical hospital information system from January 1, 2019, to December 31, 2019. Inpatient workflow, outpatient workflow, and emergency care patient workflow were modeled and used in the simulation of the logs as shown in Figure 7, Figure 8, and Figure 9, respectively. Five main modules were included in the simulation of the hospital information system: Report, Finance, Patient Management, Laboratory Management, and Pharmacy Management. In the data simulation setting, we used 19 departments and 12 roles with a total of 53 employees. The departments were information technology (3 roles), finance (1 finance officer, 3 finance support staff), administration (1 head of administration, 2 support staff), pharmacy (3 roles), and medical laboratory (5 roles). Outpatient departments included ear-nose-throat (1 doctor, 2 nurses), dentistry (1 dentist, 2 nurses), pediatric unit (1 doctor), orthopedics (1 doctor, 2 nurses), neurology (1 doctor, 2 nurses), gynecology (1 doctor, 2 nurses), endocrinology (1 doctor, 2 nurses), rheumatology (1 doctor, 2 nurses), and cancer (1 doctor, 2 nurses). The inpatient departments included patient wards and the emergency department (2 doctors, 7 nurses).

Two types of shifts were used: a regular shift and three 8-hour shifts. The regular shift is Monday to Friday from 8 AM to 4 PM, whereas the three 8-hour shifts included the following three shifts every day of the week: (1) shift 1, 6 AM to 2 PM; (2) shift 2, 2 PM to 10 PM; and (3) shift 3, 10 PM to 6 AM (next day). The numbers of roles and employees in a regular shift and in the three 8-hour shifts are shown in Table 5.

**Table 5:** Simulated departments, roles, and staff in a typical hospital.

Department		Roles (number of employees)
Information technology		Head (1), technical support (2)
Finance		Head (1), finance officer (4)
Administration		Head (1), administrative assistants (2)
Laboratory		Head (1), laboratory assistants (5)
Pharmacy		Head (1), pharmacy assistant (2)
**Outpatient**		
	Ear-nose-throat	Doctor (1), nurse (2)
	Optometry	Doctor (1), nurse (2)
	Dentistry	Doctor (1), nurse (2)
	Pediatrics	Doctor (1), nurse (2)
	Orthopedics	Doctor (1), nurse (2)
	Neurology	Doctor (1), nurse (2)
	Gynecology	Doctor (1), nurse (2)
	Endocrinology	Doctor (1), nurse (2)
	Rheumatology	Doctor (1), nurse (2)
	Cancer	Doctor (1), nurse (2)
**Inpatient**		
	Ward 1	Doctor (1), nurse (2)
	Ward 2	Doctor (1), nurse (2)
	Ward 3	Doctor (1), nurse (2)
**Three 8-hour shift**		
	Emergency	Doctor (2), nurse (2)
	Ward 1	Nurse (2)
	Ward 2	Nurse (2)
	Ward 3	Nurse (2)

Based on the flows (see Figure 6 for an example), we simulated the data and recorded the logs. The logs are considered to be normal data (nonanomaly). We also simulated some abnormal data. The abnormal data were divided into two categories: nonmalicious and malicious. Nonmalicious abnormal data were generated by simulating the “break-the-glass” scenario (eg, access by a doctor from another department due to an emergency) [2], whereas malicious abnormal data were generated by simulating attackers that are assumed to have compromised some users’ credentials and used them to access patient records (eg, identity theft). In the latter category, the attacker will access more data than legitimate users and often not follow the flows. From this data simulation, 281,886 logs were created with 273,094 normal access, 7647 nonmalicious abnormal access, and 1145 malicious access scenarios. There are 21 fields recorded in this data simulation, as displayed in Table 6.

**Table 6:** Field attributes of simulated access logs of electronic health records.

Attribute	Description
startAccessTime	The time the employee starts to access the patient record: format=day/month/year, hours:minutes:seconds
endAccessTime	The time the employee ends the patient record access: format=day/month/year, hours:minutes:seconds
employeeID	The identification number of the employee who accesses the patient record (eg, record4roleID)
roleID	The role of the employee who accesses the patient record
patientID	The identification number of the patient whose record is being accessed by the employee
activityID	The identification number of the activity (1: Create, 2: Read, 3: Update, 4: Delete)
employeeDepartmentID	The department of the employee who accesses the patient record
employeeorganizationID	The organization of the employee who accesses the patient record
osID	The operating system of the computer used by the employee to access the patient record
deviceID	The identification number of the computer used by the employee to access the patient record
browserID	The browser used by the employee to access the patient record
ipAddress	The IP address of the computer used by the employee to access the patient record
ReasonID	The reason for the employee accessing the patient record (optional)
shiftID	The identification of the shift the employee belongs to on the day of accessing the patient record
shiftStartDate	The start time of the shift the employee belongs to on the day of accessing the patient record
shiftEndDateTime	The end time of the shift the employee belongs to on the day of accessing the patient record
CRUD	The identification code of the activity (C: Create, R: Read, U: Update, D: Delete)
Access Control Status	Access control status
SessionID	The identification of the session access
AccessPatient_Warnings	Warning for unusual access
Module Used	The module accessed by the employee

### Feature Extraction

To develop the anomaly detection model, including the role classification model, some features were extracted. Each log entry represents a single transaction for a user. To analyze the user activity, the logs from each user were consolidated into a particular period. Every single activity of Doctor A would represent meaningless data points that would be difficult to analyze separately. However, by observing several activities of Doctor A for a particular period, it is easier to perform the anomaly detection task. We processed the log data into 24-hour blocks so that an instance represents the cumulative activity of a user in a single day. As a result, 25,151 instances were extracted from the raw logs, with 24,223 of them being considered normal, 585 considered nonmalicious anomaly, and 343 labeled malicious. Any access that was not for the intention of providing therapeutic functions constitutes malicious access [59]. Therefore, in the logs, malicious data represent all instances that had at least one malicious log access in a single day. The normal data represent all instances in which all of the accesses to the logs are legitimate, and the nonmalicious anomaly data represent the instances that had at least one abnormal log access, but none of them was malicious. These instances were then transformed into features for malicious access detection. Table 7 shows the features extracted from the data set.

**Table 7:** Features and their related descriptions.

Name of feature	Description
Number of create	Number of created transactions in a single day
Number of reads	Number of read transactions in a single day
Number of updates	Number of updated transactions in a single day
Number of deletes	Number of deleted transactions in a single day
Number of patient records	Number of accesses to patient records in a single day
Number of unique patients	Number of unique patients’ records accessed in a single day
Number of modules	Number of the types of modules in the information system accessed in a single day
Number of report modules	Number of transactions in the report modules in a single day
Number of finance modules	Number of finance modules accessed in a single day
Number of patient modules	Number of transactions in the patient module in a single day
Number of lab modules	Number of transactions in the laboratory module in a single day
Number of pharmacy modules	Number of transactions in the pharmacy module in a single day
Number of outside access	Number of transactions from outside the hospital network in a single day
Number of other browsers	Number of browser types used in a single day
Number of Chrome	Number of Chrome uses in a single day
Number of Internet Explorer	Number of Internet Explorer uses in a single day
Number of Safari	Number of Safari uses in a single day
Number of Firefox	Number of Firefox uses in a single day
Number of browsers	Number of other browsers used in a single day

### Performance Evaluation for Malicious Detection

For malicious access detection, several measurements, including precision, recall, and F-measures, were identified and used to evaluate the performance. All measurements were calculated based on the confusion matrix displayed in Table 8.

**Table 8:** Confusion matrix.

Actual	Predicted	
	Malicious	Nonmalicious
Malicious	True positive	False negative
Nonmalicious	False positive	True negative

True positive (TP) and true negative (TN) are the respective number of features that were correctly predicted. TP represents the malicious data that were correctly predicted as malicious, whereas TN represents the nonmalicious data that were correctly predicted as nonmalicious. False positive (FP), also often called the type I error, is the number of nonmalicious data incorrectly predicted as malicious, and false negative (FN), or the type II error, represents the malicious data incorrectly predicted as nonmalicious. The following are the formulas for each measurement:

Precision=TP/TP+FP **(1)**

Recall=TP/TP+FN **(2)**

F1=2×([precision×recall]/[precision+recall]) **(3)**

F*_β_*=(1+*β^2^*)(precision×recall)/([*β^2^*×precision]+recall) **(4)**

Equation 3 is the standard F-score formula where precision and recall have the same weight. If we want to give heavier weight to either precision or recall, we can use equation 4. For any positive real number *β*, equation 4 is the general F-measure formula where recall is considered to be more important than precision by a weight of *β* [60]. In this work, we also used the F_0.5_-score and F_2_-score. F_0.5_-score means that precision is considered to be two times more important than recall. In contrast, F_2_-score means that recall is considered to be two times more important than precision. To compute the F_0.5_-score, the *β* value was substituted with 0.5, whereas the F_2_-score was calculated by replacing the *β* value with 2.

Usually, automatic malicious behavior detection is used as a filter to narrow down the data for further manual investigation. In this case, high recall is preferred so that most of the actual malicious access will not be missed. Therefore, F_2_ is the better measure for this case. However, if we want to use the result from automatic malicious behavior detection as the final decision without further manual investigation, high precision is preferred over high recall. By using a high-precision method, almost all of the banned accesses are actually malicious. In contrast, if we use an algorithm that prefers high recall as the final decision-maker, we may ban some legitimate accesses that are mistakenly considered fraudulent. In this case, F_1_ is the better measure. However, the latter case is rarely applied in the real world since malicious behavior detection is mainly used for a decision support system before further manual investigation.

In this study, we used the logs from January to July as training data, whereas data from August to December were used for testing. The training data were used to train the role classification model, and then this model was used to detect anomalies based on the two proposed approaches. The training data contained a total of 14,558 instances with 13,977 normal instances, 339 nonmalicious anomaly instances, and 242 malicious instances. The testing data consisted of a total of 10,593 instances, with 10,246 normal instances, 246 nonmalicious anomaly instances, and 101 malicious instances.

### Experimental Results

The simulation results are summarized in Table 9 and Table 10. Table 9 shows the anomaly detection results from the first stage of two-stage malicious detection. Based on the result, the DT algorithm obtained the best precision (0.655), while the best recall was achieved by SVM (0.977). However, the best F1-score was obtained by RF (0.775). Therefore, the result that was used in the second stage was that obtained from the RF method.

**Table 9:** Anomaly detection results from the first step of two-stage malicious detection.

Classifier	Precision	Recall	F_1_
Multinomial NB^a^	0.256	0.107	0.151
Bernouilli NB	0.256	0.824	0.391
Gaussian NB	0.256	0.618	0.362
KNN^b^	0.634	0.890	0.740
NN^c^	0.651	0.941	0.770
LR^d^	0.242	0.976	0.387
RF^e^	0.662	0.934	0.775
DT^f^	0.665	0.924	0.773
SVM^g^	0.250	0.977	0.399

^a^NB: naive Bayes.

^b^KNN: k-nearest neighbor.

^c^NN: neural network.

^d^LR: logistic regression.

^e^RF: random forest.

^f^DT: decision tree.

^g^SVM: support vector machine.

Table 10 shows the malicious detection results using three approaches. The two-class approach tended to have better performance than the other two approaches. The best precision in the two-stage approach was obtained by LR with a perfect value (1.00), and KNN also had perfect precision in the three-class approach. Three classifiers (RF, DT, and SVM) in the two-class approach achieved the best precision of 0.998.

Furthermore, the best recall was obtained by NN, RF, and DT in the three-classes approach, and by Bernoulli NB and Gaussian NB in both the three-class and two-class approaches. The best F_1_ score was obtained by LR in the two-stage approach, SVM in the three-class approach, and Bernoulli NB in the two-class approach. The highest F_0.5_ score was achieved by LR, SVM, and Bernoulli NB in the two-stage, three-class, and two-class approach, respectively. Furthermore, NN and DT achieved the best F_2_ score in the two-stage approach, SVM had the best F_2_ score in the three-class approach, and Bernoulli NB had the best F_2_ score in the two-class approach. Overall, Bernoulli NB with the two-class approach achieved the best F_1_, F_0.5_, and F_2_ scores.

**Table 10:** Malicious detection results using three approaches.

Classifier			Two stage		Three classes		Two classes
**Multinomial NB^a^**							
	Precision	0.974		0.931		0.958	
	Recall	0.752		0.802		0.831	
	F_1_	0.849		0.862		0.890	
	F_0.5_	0.920		0.902		0.930	
	F_2_	0.788		0.825		0.854	
**Bernoulli NB**							
	Precision	0.977		0.824		0.997	
	Recall	0.832		0.881		0.881	
	F_1_	0.898		0.852		0.935	
	F_0.5_	0.944		0.835		0.971	
	F_2_	0.857		0.869		0.902	
**Gaussian NB**							
	Precision	0.977		0.695		0.994	
	Recall	0.832		0.881		0.881	
	F_1_	0.898		0.777		0.934	
	F_0.5_	0.944		0.726		0.969	
	F_2_	0.857		0.836		0.901	
**KNN^b^**							
	Precision	0.757		1.000		0.997	
	Recall	0.832		0.703		0.702	
	F_1_	0.792		0.826		0.824	
	F_0.5_	0.771		0.922		0.920	
	F_2_	0.816		0.747		0.746	
**NN^c^**							
	Precision	0.977		0.977		0.998	
	Recall	0.842		0.851		0.851	
	F_1_	0.904		0.910		0.919	
	F_0.5_	0.947		0.949		0.965	
	F_2_	0.866		0.874		0.877	
**LR^d^**							
	Precision	1.000		0.966		0.998	
	Recall	0.832		0.842		0.841	
	F_1_	0.908		0.899		0.913	
	F_0.5_	0.961		0.938		0.962	
	F_2_	0.861		0.864		0.868	
**RF^e^**							
	Precision	0.966		0.966		0.998	
	Recall	0.842		0.832		0.831	
	F_1_	0.899		0.894		0.907	
	F_0.5_	0.938		0.935		0.959	
	F_2_	0.864		0.855		0.860	
**DT^f^**							
	Precision	0.977		0.954		0.998	
	Recall	0.842		0.822		0.841	
	F_1_	0.904		0.883		0.913	
	F_0.5_	0.947		0.924		0.962	
	F_2_	0.866		0.845		0.868	
**SVM^g^**							
	Precision	0.988		0.978		0.998	
	Recall	0.832		0.861		0.861	
	F_1_	0.903		0.916		0.924	
	F_0.5_	0.952		0.952		0.967	
	F_2_	0.859		0.882		0.885	

^a^NB: naive Bayes.

^b^KNN: k-nearest neighbor.

^c^NN: neural network.

^d^LR: logistic regression.

^e^RF: random forest.

^f^DT: decision tree.

^g^SVM: support vector machine.

## Discussion

### Principal Findings

The main purpose of this study was to identify and assess the effectiveness of AI methods and suitable health care staff–generated security practice data for measuring the security practice of health care staff in the context of big data. The main review findings are shown in Table 11. Eighteen studies met the inclusion and exclusion criteria. Recently, a related review for countermeasures against internal threats in health care also identified five machine learning methods that were fit for such measures [30]. This suggests that the adoption of AI methods for modeling and analyzing health care professional–generated security practice data is still an emerging topic of academic interest.

**Table 11:** Principal findings of the review.

Category	Most used
Algorithms	KNN^a^ and Bayesian networks
Features	User IDs, patient IDs, device ID, date and time, location, route, and actions
Data sources	EHR^b^ and network logs
Security failures	Anomaly detection
Performance methods	True positive, false positive, false negative, ROC^c^ curve, AUC^d^
Data format	CSV^e^
Nature of data sources	Real data logs
Ground truth	Similarity measures and observed data
Privacy preserving approaches	Tokenization and deidentification

^a^KNN: k-nearest neighbor.

^b^EHR: electronic health record.

^c^ROC: receiver operating characteristic.

^d^AUC: area under the receiver operating characteristic curve.

^e^CSV: comma separated value.

### AI Methods

As shown in Tables 2 and 11, various algorithms were identified in the study, but the most used methods were KNN and NB algorithms. KNN is a supervised learning–based classification algorithm [44], which learns from labeled data. The KNN then tries to classify unlabeled data items based on the category of the majority of the most similar training data items known as K. The similarity between two data items in KNN can be determined according to the Euclidean distance of the various respective feature vectors of the data items [61]. NB is a probabilistic classifier algorithm based on the assumption that related pairs of features used for determining an outcome are independent of each other and equal [44]. There are two commonly used methods of NB for classifying text: multivariant Bernoulli and multinomial models. KNN and NB algorithms have been more commonly used based on their comparatively higher detection accuracy. For instance, in an experimental assessment of KNN and NB for security countermeasures of internal threats in health care, both models showed over 90% accuracy with NB having a slight advantage over KNN (94% vs 93%). In a related study [30], the KNN method was found to have a higher detection rate with high TP rates and low FP rates.

The major issue with KNN in the context of health care staff security–generated data is the lack of appropriate labeled data [24,53,62]. Within the health care setting, emergencies often dictate needs. In such situations, broader access to resources is normally allowed, making it challenging for reliable labeled data [24,53,62]. Therefore, in adopting KNN for empirical studies, the availability of appropriate labeled data should be considered; however, in the absence of labeled data, unsupervised clustering methods such as K-means clustering could also be considered [26].

### Input Data

The input data that were mostly used in the reviewed studies include EHR logs and network data. Yeng et al [4] analyzed observational measures toward profiling health care staff security practices, and also identified various sources, including EHR logs, browser history, network logs, and patterns of keystroke dynamics [4]. Most EHR systems use an emergency access control mechanism known as “break-the-glass” or self-authorization” [1,2]. This enables health care staff to access patients’ medical records during emergency situations without passing through conventional procedures for access authorization. A study [2] into access control methods in Norway revealed that approximately 50% of 100,000 patient records were accessed by 12,298 health care staff (representing approximately 45% of the users) through self-authorization. In such a scenario, EHR remains a vital source for analyzing deviations of required health care security practices.

Ground truth refers to the baseline, which is often used for training the algorithms [63]. The detection efficiency of the algorithms can be negatively impacted if the accuracy of the ground truth is low. As shown in Table 11, various methods—such as similarity measures, observed data, and historical methods—have been used. A similarity measure compares security practices with those of other health care professionals who have similar security practices. The observed measure is a control approach of obtaining the ground truth, whereby some users were observed to conduct their security practices under supervised, required settings [49]. However, the historical data have mainly relied on past records with a trust that the data are sufficiently reliable for the training set. These methods can be assessed for adoption in related studies.

### Features and Data Format

EHRs contain most of the features that were identified in this review, as shown in Table 3. Features such as patient ID, actions, and user ID are primary features in EHR logs. The users’ actions such as deletion, inserting, and updating, and various routes such as diagnosis, prescriptions, and drug dispensing can be tracked in EHR logs [2]. Guided with these findings, the simulated logs contained such attributes and features. Additionally, the simulation of the attributes of logs was also based on the security requirements of the EHRs of Norway [3,4,64,65]. Eventually, a total of 21 attributes and 19 features were included in the simulated logs, as shown in Tables 6 and 7, respectively.

### Security Failures and Privacy-Preserving Log Analysis

The application of AI methods to analyze big data generated by health care professional security practice is a reactive approach. With such approaches, the primary aim is to determine deviations or outliers and maliciousness in health care security practices. Anomaly in this work refers to security practices in the access logs that deviate from established security and privacy policies in accessing patient records. For instance, health care workers could be required to access patient records if the health care staff is responsible for the patient throughout their shift and for therapeutic functions. However, it becomes abnormal if the health care staff access patient records outside of their shift. Additionally, if a patient’s records are accessed when the patient has not registered for a visit to the hospital, this can also be considered abnormal. Furthermore, if health care staff are accessing patients’ records more than usual, this also raises abnormal concerns, although some anomalous access could be for therapeutic purposes and not with ill intentions. However, access that is not for therapeutic functions is described in this work as malicious. A greater proportion of the algorithms were applied for anomaly detection (67%). The detection of anomaly can clearly help in identifying the security practices that deviate from established security policies. However, Rostad and Edsberg [2] found that irregular access to patient records through self-authorization tended to be the normal security practice. An EHR system where a lot of access does not follow the established flow can make it unfeasible to manually track access with malicious intent [2]. Processing that incorporates the detection of malicious access, including intrusion detection, rather than merely detecting outliers could be an effective method of analyzing the security practice in the logs. Therefore, the identified 33% intrusion detections in the review were combined with maliciousness for the simulation since the outcome is to circumvent security requirement in both cases.

Privacy preservation in data mining provides a method to efficiently analyze data while shielding the identifications of the data subjects in a way that respects their right to privacy [66]. In the review, tokenization [43], deidentification [45], and removal of medical information [24] were some methods adopted to preserve privacy. The application of privacy-preserving methods in analyzing log data is crucial since health care data are classified among the most sensitive personal data [67]. Additionally, privacy-preserving methods need to be adopted in compliance with various regulations such as the General Data Protection Regulation [68]. Based on these findings from the review, a roadmap was drawn as a framework for empirical analysis of security practice in the big data context.

### Research Implication and Practice

In this work, a comprehensive review was performed in security practice analysis, focusing on the use of AI methods to analyze logs of health care staff. Various AI algorithms, data sources, ground truth, features, application domain data file format, and nature of data sources were identified, analyzed, and modeled. To the best of our knowledge, this is the first time such a study has been systematically performed, along with development of a model and practical assessment of the model with simulated logs for future analysis with actual health care logs. In real log analysis, essential privacy measures such as tokenization and deidentification can be adopted.

Based on the review, a concept was established (Figure 3) on how data-driven and AI methods should be adopted to analyze the logs of EHRs in security practice. The concepts (two-stage, two-class, and three-class) were implemented and their performance was assessed with simulated logs. The attributes of the logs were comprehensive based on the review, which is another major contribution of this study. In the space of supervised learning, our findings pinpoint the suitable algorithms and classification approaches that should be adopted for effective analysis of health care security practices.

Overall, the results of the simulation (Tables 9 and 10) showed that it is easier to differentiate between malicious and nonmalicious access than to distinguish between normal and nonmalicious abnormal access, which is mainly evident from the results of the two-stage approach. The performances of all classifiers in the second stage were far better than those in the first stage. This could also explain why the two-class approach was generally better than the two-stage and three-class approaches. Although the simulated data exhibited good performance with these methods, it is important to recognize that simulated data vary from real data; in particular, real data can be noisier and tend to have an adverse impact on a method’s performance [25]. In the application of real data in this framework, effective preprocessing must be carried out toward reducing the noise and its related consequences.

### Conclusion

Based on the galloping rate of data breaches in health care, HSPAMI was initiated to observe, model, and analyze health care staff security practices. One of the approaches in HSPAMI is the adoption of AI methods for modeling and analyzing health care staff–generated security practice data [4,16]. This study was then performed to identify, assess, and analyze the appropriate AI methods and data sources. Out of 130 articles that were initially identified in the context of human-generated health care data for security measures in health care, 18 articles were found to meet the inclusion and exclusion criteria. After assessment and analysis, various methods such as KNN, NB, and DT were found to have been mainly applied on EHR logs with varying input features of health care staff security practices. A framework was therefore developed and practically assessed with simulated logs based on the review, toward analyzing real EHR logs.

Based on the results, for anomaly detection, DT algorithms obtained the best precision of 0.655, whereas the best recall was achieved by SVM at 0.977. However, the best F1-score was obtained by RF at 0.775. In brief, three classifiers (RF, DT, and SVM) in the two-class approach achieved the best precision of 0.998. Moreover, for malicious access detection, LR with the two-stage approach and KNN with the three-class approach obtained perfect precision (1.00), and the best recall was obtained by Bernoulli NB and Gaussian NB in both the three-class and two-class approaches with a value of 0.881. Furthermore, the best F_1_ score, F_0.5_ score, and F_2_ score for malicious access detection were achieved by Bernoulli NB using the two-class approach with values of 0.935, 0.971, and 0.902, respectively. These methods can therefore be used in analyzing health care security practice toward finding incentive measures for information security compliance in the health care sector. This study covered only supervised learning where labeled data were used. Future work is therefore required using unsupervised learning methods in analyzing logs that do not have labeled data.

## Abbreviations

AI: artificial intelligence
CIA: confidentiality, integrity, and availability
DT: decision tree
EHR: electronic health record
FN: false negative
FP: false positive
HSPAMI: Healthcare Security Practice Analysis, Modeling, and Incentivization
LR: logistic regression
NB: naïve Bayes
NN: neural network
PRISMA: Preferred Reporting Items for Systematic Reviews and Meta-Analyses
RF: random forest
SVM: support vector machine
TN: true negative
TP: true positive
